# Nanopore sequencing identifies a higher frequency and expanded spectrum of mitochondrial DNA deletion mutations in human aging

**DOI:** 10.1111/acel.13842

**Published:** 2023-05-03

**Authors:** Amy R. Vandiver, Austin N. Hoang, Allen Herbst, Cathy C. Lee, Judd M. Aiken, Debbie McKenzie, Michael A. Teitell, Winston Timp, Jonathan Wanagat

**Affiliations:** ^1^ Division of Dermatology, Department of Medicine UCLA Los Angeles California USA; ^2^ Veterans Administration Greater Los Angeles Healthcare System Los Angeles California USA; ^3^ Division of Geriatrics, Department of Medicine UCLA Los Angeles California USA; ^4^ US Geological Survey National Wildlife Health Center Madison Wisconsin USA; ^5^ Department of Agricultural, Food and Nutritional Sciences University of Alberta Edmonton Alberta Canada; ^6^ Department of Biological Sciences University of Alberta Edmonton Alberta Canada; ^7^ Molecular Biology Institute University of California at Los Angeles Los Angeles California USA; ^8^ Department of Pathology and Laboratory Medicine, David Geffen School of Medicine University of California at Los Angeles Los Angeles California USA; ^9^ Department of Bioengineering, California NanoSystems Institute, Broad Center for Regenerative Medicine and Stem Cell Research University of California at Los Angeles Los Angeles California USA; ^10^ Department of Pediatrics, David Geffen School of Medicine University of California at Los Angeles Los Angeles California USA; ^11^ Jonsson Comprehensive Cancer Center, David Geffen School of Medicine University of California at Los Angeles Los Angeles California USA; ^12^ Department of Molecular Biology and Genetics Johns Hopkins University School of Medicine Baltimore Maryland USA; ^13^ Department of Biomedical Engineering Johns Hopkins University Baltimore Maryland USA; ^14^ Department of Genetic Medicine Johns Hopkins University School of Medicine Baltimore Maryland USA

**Keywords:** aging, DNA sequencing, human, mitochondrial DNA, skeletal muscle, substantia nigra

## Abstract

Mitochondrial DNA (mtDNA) deletion mutations cause many human diseases and are linked to age‐induced mitochondrial dysfunction. Mapping the mutation spectrum and quantifying mtDNA deletion mutation frequency is challenging with next‐generation sequencing methods. We hypothesized that long‐read sequencing of human mtDNA across the lifespan would detect a broader spectrum of mtDNA rearrangements and provide a more accurate measurement of their frequency. We employed nanopore Cas9‐targeted sequencing (nCATS) to map and quantitate mtDNA deletion mutations and develop analyses that are fit‐for‐purpose. We analyzed total DNA from vastus lateralis muscle in 15 males ranging from 20 to 81 years of age and substantia nigra from three 20‐year‐old and three 79‐year‐old men. We found that mtDNA deletion mutations detected by nCATS increased exponentially with age and mapped to a wider region of the mitochondrial genome than previously reported. Using simulated data, we observed that large deletions are often reported as chimeric alignments. To address this, we developed two algorithms for deletion identification which yield consistent deletion mapping and identify both previously reported and novel mtDNA deletion breakpoints. The identified mtDNA deletion frequency measured by nCATS correlates strongly with chronological age and predicts the deletion frequency as measured by digital PCR approaches. In substantia nigra, we observed a similar frequency of age‐related mtDNA deletions to those observed in muscle samples, but noted a distinct spectrum of deletion breakpoints. NCATS‐mtDNA sequencing allows the identification of mtDNA deletions on a single‐molecule level, characterizing the strong relationship between mtDNA deletion frequency and chronological aging.

## Graphical abstract

Cas9‐targeted nanopore sequencing of the mitochondrial genome allows for improved mapping and quantitation of age‐associated deletion mutations. This method was applied to 15 muscle and 6 substantia nigra samples from donors aged 20 to 81 years of age. These data demonstrate increased frequency and an expanded spectrum of mitochondrial genome deletion mutations and suggest tissue specific genomic localization.

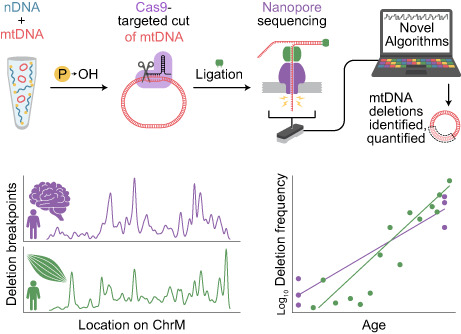

**Abbreviations**
BLAST: basic local alignment search tool
bp: basepairs
CIGAR: compact idiosyncratic gapped alignment report
ddPCR: droplet digital PCR
kbp: kilobasepairs
mtDNA: mitochodnrial DNA
nCATS: Cas9‐targeted sequencing
nDNA: nuclear DNA
NUMT: nuclear mitochondrial DNA
PCR: polymerase chain reaction

## 1 INTRODUCTION

Somatic mitochondrial DNA (mtDNA) deletion mutations are implicated in aging and age‐related diseases, but we currently lack a complete picture of the spectrum and quantity of these mutations. Age‐induced structural variants of mtDNA, such as deletions, were first reported over 40 years ago. Since that time, mtDNA deletions have been found to contribute to cell dysfunction and cell death (Baris et al., 2015; Cheema et al., 2015; Someya et al., 2008), particularly in postmitotic tissues and have been shown to be predictive of age‐induced physiological declines (Herbst, Prior, et al., 2021). Despite this importance, mapping and quantitation has been hampered by a low frequency in tissue homogenates and a corresponding need for DNA amplification. Numerous methods are available to study mtDNA deletion mutations, but these approaches are often better suited for either mapping or quantitation, rarely both. The inability to quantify and map these mutations limits both their use as potential metrics of biological aging and mechanistic investigation into their origins and consequences.

The multicopy nature and overall structure of mammalian mtDNA has important implications for the study of age‐induced structural variants. The human mitochondrial genome is 16,569 base pairs in length compared with ~6 billion base pairs in the diploid nuclear genome and is present in 10s to 1000s of copies per diploid nucleus (Anderson et al., 1981; D'Erchia et al., 2015). In high copy number tissues such as human skeletal muscle, this means mtDNA makes up about ~1% of total DNA by mass. To sequence mtDNA with sufficient coverage, studies using homogenate DNA need to account for these factors, most often through amplification or enrichment of mtDNA or removal of nDNA (Lujan et al., 2020; Zhang et al., 2012). The transposition of mtDNA sequences into nDNA (i.e., termed nuclear mitochondrial DNA segment [NUMTS]) further complicates the study of mtDNA (Albayrak et al., 2016). Structural variants may be of particular importance in the mitochondrial genome because mtDNA lacks introns and has few noncoding regions, so any structural variant is likely to disrupt gene replication, transcription, or translation. Deletion mutations, with sizes ranging from 4 to >15,000 bps, have been the most frequently studied mtDNA structural variant (Damas et al., 2014; Ruiz‐Pesini et al., 2007). A deletion event may involve flanking direct repeats, but these are not necessary for deletion formation (Fontana & Gahlon, 2020; Lee et al., 1994; Lujan et al., 2020). Numerous mechanisms are hypothesized to result in deletion formation including defects in both mtDNA replication and double‐stranded break repair (Fontana & Gahlon, 2020).

Diverse methods have been used to study and quantitate somatic mtDNA deletion mutations in aging (Lawless et al., 2020; Sanchez‐Contreras & Kennedy, 2022). Southern blot, long‐extension PCR, qPCR, and digital PCR approaches have different advantages and disadvantages, which result in differing sensitivities and specificities as well as differing reported mtDNA deletion mutation frequencies (Cao et al., 2001; He et al., 2002; Herbst, Lee, et al., 2021; Wheeler et al., 2019). In general, mtDNA deletion frequency increases with age in human muscle, but an accurate mtDNA deletion mutation frequency and spectrum remain unclear because of methodological limitations. Many indirect methods have been used to detect, sequence, and map mtDNA deletion mutations, each of which has inherent biases that limit the spectrum of mutations that can be assessed and complicate quantitation. Single‐molecule, direct DNA sequencing offers an opportunity to quantitate and map mtDNA deletion mutations without the need for mtDNA amplification, fragmentation, or enrichment. We have previously demonstrated the utility of nanopore Cas9‐targeted sequencing (nCATS) for identifying mtDNA deletions (Vandiver et al., 2022); however, our prior work focused on a small set of samples at the extremes of lifespan and used a basic analytical approach which did not detect the expected spectrum of larger deletions that have been described in aged mammals.

Here, we report our deployment of nCATS to quantify and map mitochondrial DNA deletions from otherwise healthy human skeletal muscle and substantia nigra brain tissue across the lifespan, and provide an initial analytical framework for mtDNA structural variants from long‐read sequencing data. We hypothesize that single‐molecule direct sequencing will yield a higher mutation frequency and a broader spectrum of mutations than previously reported using alternate methods. To address this, we used nCATS in total DNA from human skeletal muscle and substantia nigra and mapped deletions from long‐read DNA sequencing data. These data identify deletion mutations at higher frequencies than previously published and involving a broader genomic spectrum. Single‐molecule DNA sequencing thus provides a novel approach for quantifying aged‐induced mtDNA deletion frequency that does not require DNA amplification, fragmentation, or nDNA removal.

## 2 RESULTS

### 2.1 Optimization of deletion calling algorithm in simulated data

To identify and map mtDNA deletions in human muscle tissue, we targeted mtDNA with Cas9 targeted sequencing on Oxford Nanopore (mtDNA nCATS; Vandiver et al., 2022). In mtDNA nCATS, genomic DNA‐free ends are dephosphorylated and cas9‐guided cleavage is used to introduce new phosphorylated double‐strand breaks specifically into the mitochondrial genome. Nanopore sequencing adaptors are selectively ligated to the exposed phosphorylated ends, providing selective sequencing of mtDNA.

**FIGURE 1:**
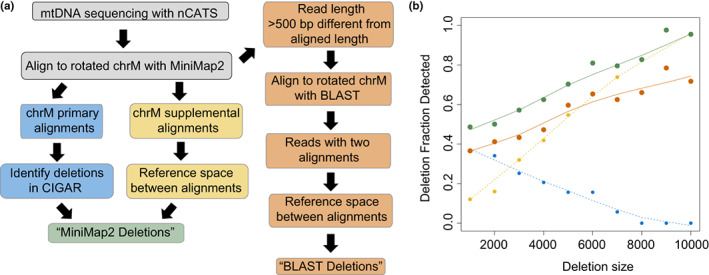
Optimization of mtDNA deletion calling algorithms using in silico test data. (a) Schematic of algorithms used for identifying large mtDNA deletions. (b) Fraction of expected deletions identified versus size of expected deletion in simulated data set. Results of primary alignment algorithm shown in blue, supplemental alignment algorithm in yellow, total MiniMap2 alignment in green, and BLAST alignment algorithm in orange.

To determine the efficacy of our initial method for identifying deletions of multiple sizes within the mitochondrial genome, an in silico test data set was generated using NanoSim (Yang et al., 2017). For this test data, sequencing reads were simulated from template genomes in which deletion events were introduced beginning at 5547 bp and extending 1, 2, 3, 4, 5, 6, 7, 8, 9 or 10 kbp with read lengths and an error profile based on our sequencing data (representative aligned reads in Figure S1). When test data were aligned with Minimap2 and deletions called based on the CIGAR sequence of the primary alignment as previously utilized to call mtDNA deletions in nanopore data (Keraite et al., 2022; Vandiver et al., 2022), there was a rapid decline in detection with increasing deletion size (Figure 1b). Closer examination of aligned data demonstrated that many reads containing large deletions were aligned as chimeric alignments with a “primary” alignment and a nonoverlapping “supplemental” alignment. To address this, we developed an algorithm to identify deletions between chimeric alignments from Minimap2 (Figure 1a) that are nonoverlapping and contiguous on the query sequence but distant on the reference sequence. Using this approach, we noted increased detection of larger deletions in simulated data (Figure 1b).

To determine whether our deletion calling approach was dependent on the choice of aligner, we utilized a parallel method in which chromosome M reads for which query length diverged from aligned length by >500 bp were realigned using BLAST. If this returned two nonoverlapping alignments that were contiguous on the query sequence but distant on the reference sequence, a deletion was identified as the space between the two alignments (Figure 1a). Using this method on our simulated data, we observed decreased sensitivity in detecting deletions (Figure 1b), but a similar performance in relationship to deletion size.

### 2.2 Long‐read sequencing of mtDNA identifies mtDNA deletion breakpoints in muscle samples

We applied mtDNA nCATS to 15 human muscle samples obtained from male donors ranging from 26 to 81 years of age (Table S1). Prior to sequencing, the mtDNA copy number and frequency of large deletions in each sample were quantified using our validated droplet digital PCR assay (Herbst, Lee, et al., 2021), which indicated a 1.7‐fold decrease in copy number from youngest to oldest sample and logarithmically increasing frequency of large deletions with increasing donor age (Figure S2). Nanopore sequencing generated an average of 162,947 reads per sample, with a range of 64%–85% of reads aligned to the reference mitochondrial genome. Between 82% and 90% of reads aligned to the forward strand of our reference genome, in the direction downstream of the Cas9 PAM site.

**FIGURE 2:**
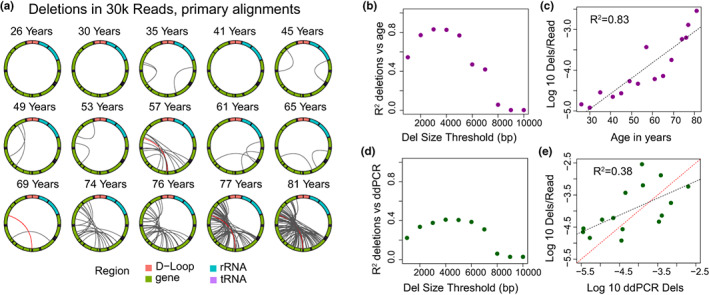
Large mtDNA deletions in primary alignments. (a) Localization of deletions >2000 bp identified in CIGAR sequence of primary alignments in a random subset of 30,000 reads per sample. The mitochondrial genome is depicted in a clockwise orientation with lines linking the start and end of each identified deletion. The human mitochondrial “common” deletion is highlighted in red where identified. (b) *R* ^2^ correlation coefficient for log‐transformed number of identified deletions per mitochondrial read versus age plotted versus the minimum deletion size cutoff used in calculating correlation. (c) Log‐transformed frequency of deletions >3 kbp per mitochondrial read versus age. (d) *R* ^2^ correlation coefficient for log‐transformed number of identified deletions per mitochondrial read versus log‐transformed deletion frequency by ddPCR plotted versus the minimum deletion size cutoff used in calculating correlation. (e) Log‐transformed frequency of deletions >3 kbp per mitochondrial read versus log‐transformed ddPCR deletion frequency. The red dashed line is the line of identity.

Deletions were first identified through parsing of the CIGAR sequence from the primary alignment of each read as reported previously (Keraite et al., 2022; Vandiver et al., 2022). We identified a total of 10,712 deletion events >100 bp across all 15 samples, ranging from 101 to 8131 bp. The previously identified mitochondrial “common deletion,” a deletion event spanning 8470–13,477 bp, which is associated with Kearns–Sayre syndrome and frequently reported in aged tissue (Meissner et al., 2008), was identified 51 times, representing 5.7% of deletions >2 kb identified (shown in red in Figure 2a). To normalize for differences in read depth in visualizing data, we selected a random subsample of 30,000 reads from each sample for plotting (Figure 2a). Next, to understand the frequency of deletions per molecule of mtDNA, we calculated the frequency of deletions of specific minimum sizes per mtDNA read. To determine what minimum threshold for deletion size most significantly correlates with chronological age, we calculated the correlation coefficient between deletion frequency and chronological age using a range of minimum deletion sizes (Figure 2b). The highest correlation to chronologic age was observed using a minimum deletion size of 3 kbp (*R* ^2^ = 0.83, Figure 2c). We noted a steep drop off in correlation between deletion frequency and age when deletion size thresholds >5 kbp were considered, which is likely related to the decreased sensitivity of this algorithm for detecting this size of deletion (Figure 1b). To understand the relationship between our calculated deletion frequencies and droplet digital PCR quantifications of deletion frequency, we calculated the correlation coefficient between nanopore deletion frequency and droplet digital PCR deletion frequency for each sample using a range of minimum deletion sizes (Figure 2d). The abundance of deletions >3 kbp per read detected using nCATS was correlated with the log‐transformed deletion frequency as obtained by droplet digital PCR (*R* ^2^ = 0.38, Figure 2e).

**FIGURE 3:**
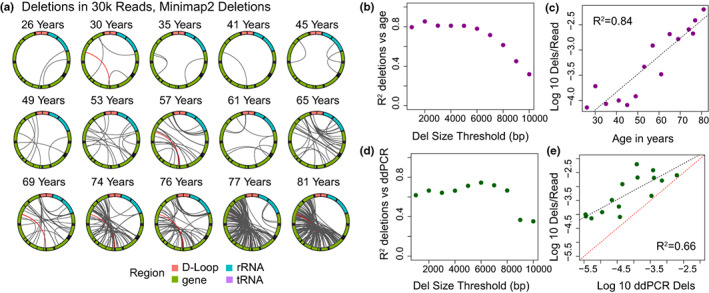
Large mtDNA deletions mapped using combined Minimap2 alignments. (a) Localization of deletions >2000 bp identified using the Minimap2 algorithm in a random subset of 30,000 reads per sample. The mitochondrial genome is depicted in a clockwise orientation with lines linking the start and end of each identified deletion. The human mitochondrial “common” deletion is highlighted in red where it was detected. (b) *R* ^2^ correlation coefficient for log‐transformed number of identified deletions per mitochondrial read versus age plotted versus the minimum deletion size cutoff used in calculating correlation. (c) Log‐transformed frequency of deletions >2 kbp per mitochondrial read versus age. (d) *R* ^2^ correlation coefficient for log‐transformed number of identified deletions per mitochondrial read versus log‐transformed deletion frequency by ddPCR plotted versus the minimum deletion size cutoff used in calculating correlation. (e) Log‐transformed frequency of deletions >2 kbp per mitochondrial read versus log‐transformed ddPCR deletion frequency. Red dashed line is the line of identity.

Given the increased ability to identify larger deletions when considering chimeric alignments, we next applied the algorithm optimized on simulated data to identify deletions using Minimap2 chimeric alignments and combined these with the deletions identified in primary alignments (Figure 1a). In order to avoid contamination from nuclear DNA sequence, we limited this analysis to reads >400 bp, the minimum for accurate mtDNA mapping in 100% of bases defined by prior analyses (Albayrak et al., 2016), and <17,000 bp, above which may raise concern for nuclear contamination. We further filtered out reads in which chimeric alignments that were distant on the query sequence, as these may represent alignment to nonmitochondrial sequence. With this combined set, we identified a total of 12,621 deletion events with sizes ranging from 138 to 14,146 bp (deletions >2 kbp in a subset of 30k reads per sample shown in Figure 3a). In the total set, the “common deletion” was detected 100 times, representing 4.18% of deletions >2 kbp. The strongest correlation (*R* ^2^ = 0.84) of deletions per read to chronological age was observed when using a minimum deletion size threshold of 2 kbp (Figure 3b,c). We noted a less steep decline in correlation coefficient at larger deletion size thresholds than when only primary alignments were considered, consistent with the improved ability to detect larger deletions. However, we observe a persistent low correlation between age and deletion frequency when only the largest deletions are considered, which is likely due to lower number of events in this size range, particularly at younger age.

**FIGURE 4:**
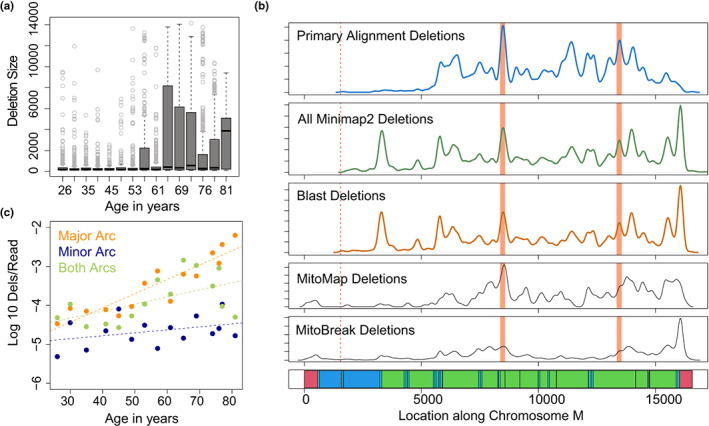
Distribution of mtDNA deletions across the mitochondrial genome. (a) Boxplots depicting the distribution of mtDNA deletion size versus age of sample. (b) Density of deletion breakpoints identified using primary alignment algorithm (blue), combined Minimap2 deletions (green) and blast alignment deletions (dark orange) as compared to all deletion breakpoints reported in MitoMap (Lott et al., 2013) and MitoBreak (Damas et al., 2014) databases. Bottom panel shows annotation of the mitochondrial genome. D‐loop regions shown in red, rRNA shown in dark blue, tRNA shown in light blue, coding regions shown in green. Cas9 cut site shown in dotted lines. Location of human common deletion breakpoints shown in light red rectangles. (c) Log‐transformed frequency of deletions >2 kbp per mitochondrial read versus age, plotted separately for deletions contained entirely within the minor arc (dark blue) entirely within the major arc (orange), or involving both arcs (green).

Using a minimum deletion size of 2 kbp, regression averages to a deletion frequency of 4.3 × 10^−5^ for 25‐year‐old individuals and increases 59‐fold to 2.5 × 10^−3^ for 75‐year‐old individuals. The frequency of deletions per mtDNA read correlates strongly with ddPCR deletion frequency (*R* ^2^ = 0.66), with a higher deletion frequency noted in sequencing data than ddPCR at all ages (Figure 3e). In addition to changes in deletion frequency, the mean size of deletions was observed to increase significantly with age (*R* ^2^ = 0.60, *p* < 0.01; distribution shown in Figure 4a). Comparable results were obtained when we applied the BLAST deletion calling algorithm. Using this approach, we detected a total of 10,810 deletions events ranging from 101 to 14,045 bp across all samples (deletions >2 kbp in a subset of 30k reads per sample shown in Figure S3a). A strong correlation (*R* ^2^ = 0.66) between deletion frequency and age (Figure S3b,c) and deletion frequency and ddPCR deletion frequency was also observed using this approach (Figure S3d,e).

In order to further evaluate the potential for NUMT contamination in our sequences, we utilized an established tool for assessing mitochondrial haplogroup group contamination in sequencing data, Haplocheck (Weissensteiner et al., 2021). When applied to all reads, no evidence of mtDNA haplogroup contamination was found in any sample (Table S2). To further evaluate for potential enrichment of NUMTs specifically in reads containing deletions, Haplocheck was next applied to only reads identified as containing a deletion from each sample. This showed no evidence of mtDNA haplogroup contamination.

### 2.3 Long‐read sequencing maps mtDNA deletions across mitochondrial genome

The ability to directly map deletion breakpoints in a less biased manner with nCATS‐mtDNA sequencing provides new opportunities to understand the distribution of mtDNA deletion breakpoints across the mitochondrial genome. To begin understanding these data, we compared the distribution of deletion breakpoints identified using our methods to deletion breakpoints identified through other methods in the Mitomap (Lott et al., 2013) and MitoBreak (Damas et al., 2014) databases (Figure 4b). Utilizing a permutation test of equality through the *sm* package in R, deletion breakpoints identified in primary alignments were not significantly different from deletion breakpoints identified in both Mitomap and Mitobreak (*p* = 0.70, *p* = 0.24). When Minimap2 chimeric alignments were also considered, the breakpoint distribution was significantly distinct from deletion breakpoints identified in Mitomap (*p* = 0.03) but not from those identified in MitoBreak (*p* = 0.52). Notably, no significant difference was identified between deletion breakpoints identified using Minimap2 and Blast alignments (*p* = 0.93, Figure 4b). Intriguingly, while deletions within the “minor arc” spanning 408–5746 bp were noted, the frequency of deletions identified entirely within the minor arc did not change significantly with age (linear *R* ^2^ = 0.13, *p* = 0.2), while the frequency of deletions entirely within the major arc correlates strongly with age (*R* ^2^ = 0.83, *p* < 0.001). Deletions involving both arcs, which account for 6.5% of all deletions, also correlate significantly with age (*R* ^2^ = 0.30, *p* = 0.03). This pattern was observed considering deletions >2 kbps (Figure 4c) and deletions of all sizes (Figure S4).

### 2.4 Large mtDNA deletions increase with age in human substantia nigra

**FIGURE 5:**
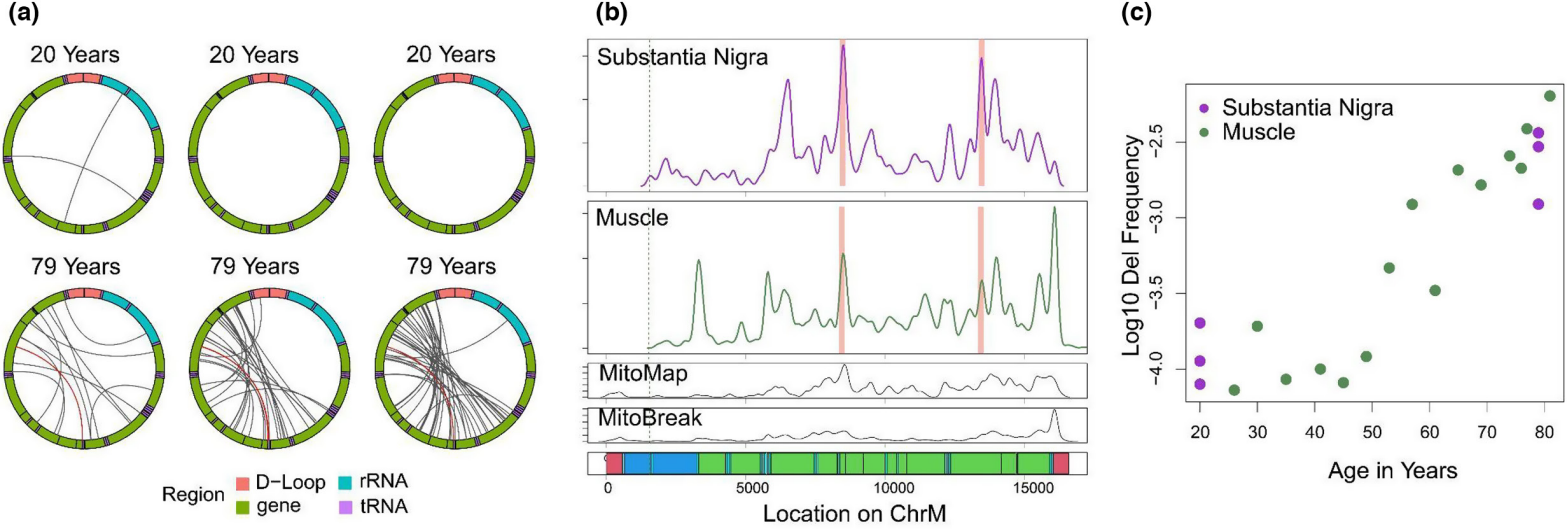
Mapping of large mtDNA deletions in human substantia nigra. (a) Localization of deletions >2000 bp identified using minimap2 alignments in a random subset of 12,000 reads per sample. The mitochondrial genome is depicted in a clockwise orientation with lines linking the start and end of each identified deletion. The human mitochondrial “common” deletion is highlighted in red where identified. (b) Density of deletion breakpoints identified in human substantia nigra (top panel), as compared to those identified in human muscle tissue (second panel), the MitoMap database (third panel) and the MitoBreak database (fourth panel). Bottom panel shows the annotation of the mitochondrial genome. D‐loop regions shown in red, rRNA shown in dark blue, tRNA shown in light blue, coding regions shown in green. Cas9 cut site shown in dotted lines. Location of human common deletion breakpoints shown in light red rectangles. (c) Log 10‐transformed frequency of mtDNA deletions >2000 bp in total chromosome M reads vs age for muscle (green) and substantia nigra (purple) samples.

To assess the applicability of our algorithm to a nonmuscle tissue with known age‐related mtDNA changes, we next used nCATS to obtain long‐read sequencing of mtDNA from human substantia nigra. For this analysis, we sequenced DNA isolated from substantia nigra from three 20‐year‐old male donors and three 79‐year‐old male donors. Nanopore sequencing generated a mean of 123,784 reads per sample, with a range of 9%–75% of reads aligned to the reference mitochondrial genome. In these samples, we determined the location of mtDNA deletions using Minimap2 alignments. Using this analysis, we identified a total of 1945 deletion events, ranging from 101 to 13,909 bp (Deletions >2 kbp identified in a subset of 12k reads per sample shown in Figure 5a). We noted a significantly increased number of deletions >2 kbp per read in the 79‐year‐old as compared to 20‐year‐old donors (Welch's *t*‐test, *p* = 0.038). We next examined the distribution of deletion breakpoints identified in substantia nigra samples as compared to muscle samples and the published databases using a permutation test of equality. A significant difference was noted between the distribution of deletion breakpoints identified in muscle and substantia nigra samples (permutation testing, *p* < 0.01) with a decrease in deletion breakpoints identified at tRNA‐T (bp position 15,888–15,953) in substantia nigra tissue (Figure 5b). Despite this difference in distribution, the frequency of mtDNA deletions per mtDNA read was similar between substantia nigra and muscle samples at each age time point (Figure 5c). All deletion breakpoint locations are provided in Data S1.

Next, to test the utility of our method on a tissue with low expected mtDNA deletion frequency, we utilized nCATs to obtain long‐read mtDNA sequencing from two human placenta samples. Nanopore sequencing generated an average of 79,644 reads per sample, with 24.4% and 38.7% of reads aligned to the reference mitochondrial genome. Using Minimap2 alignments, only 7 deletions >2 kbp were detected across both samples, consistent with the expected low level of age‐related mtDNA change (Figure S5).

## 3 DISCUSSION

In multiple tissues across the human lifespan, we deploy nCATS (nanopore Cas9‐targeted sequencing) to provide an amplification‐free mapping and quantitation of age‐induced mtDNA deletion frequency. Using this approach and a novel mapping algorithm to address chimeric alignments, we find mtDNA deletion mutation frequencies that are higher than generally reported for aging human skeletal muscle, an expanded scope of deletion breakpoints and a tissue‐specific spectrum of deletions. We observe that the mapping algorithms used have important effects on the spectrum of deletions called and their quantitation. Finally, we demonstrate an age‐associated shift in the deletion events detected: the mean length of deletions detected increases with age of sample and the proportion of deletions detected within the minor arc decreases.

These findings expand our current understanding of age‐induced mtDNA deletion mutations. Many different approaches have been used to detect and quantitate mtDNA deletion mutations including Southern blot, PCR, qPCR, long‐extension PCR, digital PCR, and NGS (Cao et al., 2001; He et al., 2002; Herbst, Lee, et al., 2021; Lujan et al., 2020; Wheeler et al., 2019). Because of the excess of nuclear DNA in total DNA samples, most approaches to mtDNA deletion mutation detection leverage some type of enrichment, including mitochondrial isolation (Devall et al., 2015), nuclease digestion of nDNA (Lujan et al., 2020; Yao et al., 2019), and preferential amplification of mtDNA (Zhang et al., 2012), each of which comes with biases. The nCATS approach avoids DNA amplification, decreasing some of these biases and allowing new insights. In our work, we find up to ~10‐fold higher mutation frequencies using the nCATS approach and a strong, exponential correlation between mutation frequency and age, demonstrating the relevance of the identified changes. These data support our hypothesis that the nCATS approach detects an expanded population of deletion events.

One benefit of long‐read sequencing is the decreased likelihood of erroneously calling mtDNA variants from nuclear DNA of mitochondrial origin (NUMTs), which has complicated short‐read sequencing‐based analysis of mtDNA variation (Wei et al., 2020). Our method selectively sequences mtDNA using Cas9‐guided cutting of mtDNA. While there is the potential for off‐target cutting in the nuclear genome with our chosen guide sequence, this would introduce only a single cut in the nuclear chromosomes, generating sequences dramatically longer than 16.5 kbp; thus, we only considered deletions from reads shorter than 17 kbp. Previous studies examining the potential for nuclear DNA to cause false identification of mtDNA heteroplasmy indicate the vast majority of regions of identical or near identical sequence are <417 base pairs, and recommend that to avoid nDNA interference in 100% of mtDNA bases, reads should be longer than 417 base pairs (Albayrak et al., 2016); thus, we only considered deletions from reads above this length. Other work has indicated the presence of longer NUMTs, approaching the full length of the mitochondrial genome, which may be polymorphic between individuals (Dayama et al., 2014), which thus remains a theoretical concern for calling low‐frequency heteroplasmy using our sequencing method; however, we are unaware of reports of NUMTs containing large structural rearrangements. Should such NUMTs exist and bypass our size cutoffs, these could potentially contribute a maximum of one deletion out of ~4000 mtDNA reads (the average mtDNA copy number in our samples), over an order of magnitude less than observed with age‐related change.

Our findings offer intriguing parallels and contrast to the recent comprehensive mtDNA deletion analysis from short‐read data from skeletal muscle (Lujan et al., 2020). In that work, a large number of deletions are inferred in aged muscle; however, the use of short‐read sequencing limits the ability to calculate a deletion frequency, so the measures cannot be directly compared with droplet digital PCR, or to our nCATS data. As an intriguing parallel, Lujan et al. observed deletions involving both the minor and major arcs with a relative few crossing the light strand origin of replication to involve both arcs, similar to the distribution observed in our work. We address the relationship between deletion frequency in each location and donor age as well as the tissue specificity of deletion distribution, which was not explored by Lujan et al. Our work expands upon their findings by offering orthogonal approaches without some of the limitations of short‐read sequencing and increased insight into the specific deletions correlated with age in muscle and brain tissue.

Detecting a larger population of deletion events also reveals a broader spectrum of mtDNA deletion mutation breakpoints. In previous studies, breakpoints were observed predominantly within the mitochondrial major arc spanning from base pair 5747 to 407 (Eimon et al., 1996), although breakpoints spanning both major and minor arcs (Bua et al., 2006) and entirely within the minor arc have also been reported (Lujan et al., 2020). One major arc deletion, known as the mitochondrial “common deletion,” has been frequently reported in tissue homogenate studies, but has not been detected in single muscle fiber or single‐cell studies. In our work, we observe the presence of the common deletion as well as many of the previously reported deletion breakpoints, validating the utility of our method for breakpoint detection. However, in addition to observation of these previously reported events, we observe novel deletion breakpoints, including those within the mitochondrial minor arc. This finding leads to two intriguing observations: First, we note that while minor arc deletions are present, their frequency does not increase with age as seen with major arc deletions, suggesting possible different mechanisms for development and amplification of minor versus major arc deletions. Second, we note a distinct distribution of deletion breakpoints in substantia nigra versus muscle tissue samples, indicating possible tissue specificity in the mechanism of deletion formation or amplification. While these findings have potential impacts for the study of mtDNA deletions, we note multiple limitations to the current study. Regarding the identification of minor arc deletions, these are by nature smaller than deletions involving the major arc and thus may be more affected by the lower sensitivity of our algorithm for smaller deletions. We thus recognize that a full understanding of the intricacies of deletion distribution with age and between tissues will require larger sample sizes across the lifespan and cross‐validation between multiple modalities.

This work demonstrates the potential of nCATS‐mtDNA for mapping and quantitating age‐induced deletion mutations, laying a foundation to deepen the understanding of these mutations in human aging and relevant clinical settings. We note that the current work is limited in scope and thus does not fully elucidate the broad potential for studying human aging. Specifically, this initial study was limited to male samples and only 15 skeletal muscle and 6 substantia nigra samples. We have only male subjects at this point due to our focus on physical function with age in older US veterans, who are majority male. MtDNA deletion mutation frequency, on average, is lower in women than in men (Herbst, Prior, et al., 2021) using dPCR methodology. We hypothesize that nanopore sequencing will reveal similar sex differences in skeletal muscle deletion frequency across the human lifespan. A benefit of our current sample set is the orthogonal ddPCR data from the same samples, which add rigor to our findings of age‐induced increases in mtDNA deletion mutation frequency in human muscle. The second main limitation is the lack of established analysis algorithms for mtDNA deletion detection in long‐read sequencing. The algorithms presented here represent first efforts in aligning and mapping mtDNA structural variants. We expect that emerging data on long‐read mtDNA sequencping will accelerate the development and validation of improved algorithms for these analyses, akin to the continued evolution of pipelines to detect such variants in NGS data (Basu et al., 2020; Bosworth et al., 2017). The third main limitation is the use of a single cut site located in the minor arc for mtDNA cleavage. The selection of this single site was based on our current understanding of the known distribution of age‐ and disease‐induced mtDNA deletion mutations (Bua et al., 2006; Damas et al., 2014). The data from this study demonstrate that a broader spectrum of mtDNA deletions exists and, thus, using different cut sites may further alter the deletion breakpoint distribution in future studies.

To further the utility of mtDNA nCATS for use in human studies, future work will focus on expansion and diversification of the data set, including increased sample numbers, tissue types, and female donors as well as continued optimization of analytical methods. The data generated will continue to serve as a template for the optimization of analytical pipelines to streamline mtDNA deletion mutation mapping. Applying mtDNA nCATS to more human tissues and including samples from both sexes will extend the relevance and generalizability of the data set. Optimization of the experimental and analytical pipelines would also afford opportunities for more formal validation of performance characteristics for nCATS including limit of detection, precision, and specificity (Burd, 2010). Here, we demonstrate that long‐read, direct sequencing of human mtDNA enhances the detection and quantitation of age‐induced mtDNA deletion mutations. These data indicate that there is more to learn regarding the frequency and spectrum of these mutations in aging tissues.

## 4 CONCLUSIONS

NCATS‐mtDNA sequencing allows the identification of mtDNA deletions on a single‐molecule level, characterizing the strong relationship between mtDNA deletion frequency and chronological aging in multiple tissues. These data offer new insight into the frequency and distribution of age‐associated mtDNA deletions, providing a foundation for further study into the mechanism of mtDNA deletion formation and accumulation and the use of mtDNA deletions as a metric of tissue aging.

## 5 MATERIALS AND METHODS

### 5.1 Human subjects

Deidentified muscle biopsy specimens were collected as part of a VA Merit Award, “Testosterone, inflammation and metabolic risk in older Veterans” and NIH R01DK090406 (PI: Cathy C. Lee, MD). Use of the human specimens for this study was approved by the UCLA Institutional Review Board (Protocol #18‐001547) and the University of Alberta Health Research Ethics Board #00084515. The biopsy samples were obtained from the vastus lateralis muscle of 15 male subjects ranging in age from 20 to 81 years (Lee et al., 2020). Personnel analyzing human muscle biopsy samples were blinded to subject age. Subject ages were only revealed after analyses were completed. Substantia nigra samples were obtained from the NIH NeuroBiobank, and all subjects were men with no known neurologic disease.

### 5.2 DNA isolation and quality control

Tissue samples were powdered under liquid nitrogen using a mortar and pestle. Approximately 25 mg of powdered muscle was used for DNA isolation, performed by proteinase K and RNase A digestion, phenol/chloroform extraction, and ethanol precipitation, as previously described (Herbst et al., 2017). Quality control of the DNA was performed via Nanodrop (Nanodrop 2000; Thermo Scientific), Qubit (2.0; Invitrogen), and gel electrophoresis. Quality control cutoffs for total DNA included A260/280 > 1.8; A260/230 > 1.8.

### 5.3 Cas9 cleavage, library preparation, and nanopore sequencing

A custom guide RNA sequence targeting human mtDNA: ACCCCTACGCATTTATATAG was used in accordance with previously described methods (Vandiver et al., 2022). Precomplexed Alt‐R CRISPR‐Cas9 single‐guide RNA (IDT) was diluted to a concentration of 10 μM and combined with HiFi Cas9 Nuclease V3 (IDT, cat 1081060) in CutSmart Buffer (NEB, cat B7204).

Cas9 cleavage and library preparation was performed in accordance with previously described methods (Gilpatrick et al., 2020). Approximately 3 μg of input DNA was dephosphorylated with alkaline phosphatase (ONT, cat SQK‐CS9109) in CutSmart Buffer (NEB, cat B7204). Following enzyme inactivation of alkaline phosphatase, 10 μL of 333 nM Cas9‐gRNA complex was combined with the dephosphorylated DNA, dATP (ONT, cat SQK‐CS9109), and Taq DNA polymerase (ONT, cat SQK‐CS9109). After Cas9 cleavage and dA‐tailing, sequencing adaptors were ligated to target DNA ends and library purification was performed using the Oxford Nanopore Technologies Cas9 Sequencing Kit (ONT, cat SQK‐CS9109). Samples were run on a MinION flow cell (v9.4.1) using the MinION Mk1C sequencer. Sequencing and basecalling was operated using the MinKNOW software (v21.10.8).

### 5.4 Sequence, structural rearrangement analysis, and statistics

Analysis was done using python version 3.6.15 and R version 4.0.2.

#### 5.4.1 Reference genome

The chromosome M reference from HG38 was rotated by removing the base pairs prior to our cut site (1:1547) and concatenating these to the end of the reference sequence in order to account for the expected start and end of our sequenced DNA and prevent aberrant deletion calling.

#### 5.4.2 Base calling

Base calling was performed using Guppy (v4.5.4, release 4/20/21) on MinKnow (v4.2.8). Reads meeting a QC threshold of 5 were included in analysis.

#### 5.4.3 Alignment

All sequences passing quality control were aligned to the rotated mitochondrial genome using Minimap2 (Li, 2018), version 2.24.

#### 5.4.4 Simulated data

Test data were generated using NanoSim (Yang et al., 2017; version 3.0.2). A representative run of our muscle data was used as a reference for read profile generation to identify read lengths and quality parameters on which simulated data were based. Five hundred reads were simulated from 10 different template genomes. Each genome contained a deletion beginning at 4000 bp of the rotated genome, extending either 1, 2, 3, 4, 5, 6, 7, 8, 9, or 10 kbp. After read generation, all 5000 reads were combined for subsequent alignment and deletion calling in order to generate a simulated data set with an even distribution of deletions of each size.

#### 5.4.5 Deletions in primary alignment

Deletions were identified from primary alignments by parsing the CiGAR sequence of each ChrM primary alignment using GenomicAlignments package in R (version 1.26.0; Lawrence et al., 2013) to identify deletions >100 bp. Deletions within 300 bp were combined to a single deletion event.

#### 5.4.6 Deletions in supplemental alignments

Alignments to ChrM with the 2048 flag were extracted. Positional information and span across the reference genome for each supplemental alignment and the paired primary alignment was determined using RSamtools (version 2.6.0). For reads <17,000 bp in which the primary and supplemental alignments were each >200 bp and were contiguous within 300 bp on the query sequence but distant on the reference sequence, and primary and supplemental alignments overlap by <50 bp on query and reference sequences, deletions were identified as the position between the primary and supplemental alignments on the reference genome.

#### 5.4.7 Deletion calling using BLAST

For reads with primary alignment to ChrM, the total read length was compared with the length of the primary alignment on the reference genome using RSamtools. For reads in which the read length was >500 bp shorter or longer than the aligned length, the sequence was realigned to the rotated reference genome using BLAST (Basic Local Alignment Search Tool) aligner via rBLAST (version 0.99.2). For reads <17,000 bp with two nonoverlapping alignments in BLAST that were each >200 bp and were contiguous within 300 bp on the query sequence but distant on the reference sequence, deletions were identified as the position between the alignments.

#### 5.4.8 Density analysis

Distribution of deletion breakpoints across the mitochondrial genome was compared using a permutation test of equality through the sm package (version 2.2‐5.7) with a smoothing parameter of 50.

#### 5.4.9 Correlation analysis

Linear regression was performed using base R methods. Multiple *R*‐squared values are reported.

Analysis code is available at https://github.com/amyruthvandiver/Nanopore_Tissues.

## AUTHOR CONTRIBUTIONS

This study was designed and conceived by AV, ANH, and JW. Sample preparation was performed by ANH. Data analyses were performed by AV. All authors contributed to writing and revising the manuscript.

## FUNDING INFORMATION

This work is supported by the National Institute on Aging at the National Institutes of Health (grant numbers R56AG060880, R01AG055518, K02AG059847, and R01AG069924) and the National Institute of Arthritis and Musculoskeletal and Skin Diseases (T32 AR071307) and The Dermatology Foundation. This material is the result of work supported with resources and the use of facilities at the Veterans Administration Greater Los Angeles Healthcare System.

## CONFLICT OF INTEREST STATEMENT

Dr. Timp holds two patents (US 8,748,091 and US 8,394,584) which have been licensed by Oxford Nanopore Technologies.

## CONSENT TO PARTICIPATE

Deidentified muscle biopsy specimens were collected as part of a VA Merit Award, “Testosterone, inflammation and metabolic risk in older Veterans” and NIH R01DK090406 (PI: Cathy Lee, MD).

## Supporting information


Figures

TableS1

TableS2

DataS1

## Data Availability

The data that support the findings of this study are available from the corresponding author upon reasonable request and through SRA, under Bioproject PRJNA945898. Analytical code is available at https://github.com/amyruthvandiver/Nanopore_Tissues. Archived lists of previously reported deletion breakpoints from MitoMap are available at: https://www.mitomap.org/foswiki/bin/view/MITOMAP/DeletionsSingle and from MitoBreak are available at: http://mitobreak.portugene.com/cgi‐bin/Mitobreak_showtable.cgi?Species=Homsap&Breaktype=Del&Published=Pub.
