# Supplementary material for: Nanopore sequencing identifies a higher frequency and expanded spectrum of mitochondrial DNA deletion mutations in human aging
Source: Aging Cell. 2023 May 3;22(6):e13842. doi: 10.1111/acel.13842 (PMC10265159; doi:10.1111/acel.13842)
Supplement: Supplementary file 3 — TableS2 [file ACEL-22-e13842-s004.zip › ACEL_13842_Supplementary table 2 captions.docx]

The title for Supplementary Table 2 should be "Haplocheck Analysis".
